# Evaluating hospital information systems from the point of view of the medical records section users in Medical-Educational Hospitals of Kermanshah 2014

**Published:** 2015

**Authors:** S Rostami, A Sarmad, M Mohammadi, M Cheleie, S Amiri, SH Zardoei Golanbary

**Affiliations:** * Medical Record, Kermanshah University of Medical Sciences, Kermanshah, Iran; ** Health Information Technology, School of Allied Medical Sciences, Tehran University of Medical Sciences, Tehran, Iran; *** Health Service Management, Kermanshah University of Medical Sciences, Kermanshah, Iran; **** Health Professional, Kermanshah University of Medical Sciences, Kermanshah, Iran

**Keywords:** hospital information system, evaluation, medical records section employees, isometric measures

## Abstract

Evaluating hospital information systems leads to the improvement and devotion based on the users’ needs, especially the medical records section users in hospitals, which are in contact with this system from the moment the patient enters the hospital until his/ her release and after that. The present research aimed to evaluate the hospital information systems from the point of view of the medical record section employees.

**Materials and method**:

The current research was applicative-descriptive analytical and the research society included 70 users of the medical history section in the educational-medical centers of Kermanshah city. The data-gathering tool was the 10th part of 9241/ 10 Isometric standard questionnaire of evaluating hospital information systems, with 75 specific questions in 7 bases, with the five spectra Likertt scale, its conceptual admissibility being confirmed in previous researches. 22 SPSS statistical software analyzed its permanency in the present study, which was also confirmed by Cronbach’s’s alpha test, which equaled to 0.89, and the data.

**Findings**:

The highest level of the employees’ satisfaction, based on gained scores median, was respectively the incompatibility with the users’ expectations, measuring 3.55, self-description measuring 3.54 and controllability - 3.51, which in total presented the average scores of 3.39, the lowest level of satisfaction being related to useful learning , whose value was 3.19.

**Discussion and conclusion**:

Hospital information systems’ users believe that it is more desirable that the existing systems are based on the measures and consider them proper for making them non-governmental and useful for undesired learning. Considering the long distance of the existing information systems with the desired performance, it is essential that “these systems pay more attention to a more complete and deeper recognition and awareness of users’ opinions and requirements in their road. The movement and development is to increase their chance in succeeding and achieving their goals, where the goal is to improve the patients’ care and improve the health of the society members with the help of information technology.

## Introduction

The volume of information produced in hospitals that transfers from a section to another is rather considerable [**3**]. A hospital information system is the general software for the integration of the data related to a patient, sending and exchanging patient’s general information among various hospital sections and other medical centers to speed up the patient’s treatment and therapy process, improve the quality, increase the satisfaction, and decrease the expenses [**4**]. These systems are the complete sample for the reflection of the hospital’s image to clinical and executive managers [**5**]. However, the information about the real advantages and disadvantages of such systems for the informed purchase of such technology are reachable [**6**]. If a hospital’s information system were not compatible with the individual’s general duties, using it would be complicated and might be hard to understand. The users will not like it and in case it does not satisfy the needs, the users would ignore it, and they even might consider the system an intruder and irritating factor [**7**]. Evaluating health information systems is one of the methods to increase the reliability coefficient of these systems’ efficiency [**8**]. On one side, it is critical to assess these systems’ performance. Evaluating the health systems means the act of measuring or identifying a health information system that leads to taking required operations decisions about a particular issue [**9**]. 

Considering the extended effect of information technology in organizations and expenses related to information systems, there has been an increasing need for the evaluation of the quality of these services, primarily assessing the satisfaction felt by users [**10**]. Evaluating the hospital information system leads to improving and developing it properly to users’ requirements and increasing the effectiveness and efficiency of the hospital. Identifying the users’ point of view regarding the quality and efficiency of the systems is critical and affects the development and accomplishment of the scheme. The universal health organization believes that primarily the evaluation to determine the weak and strong points of the system in presenting services should be done, also identify its disadvantages, and give general suggestions for corrective actions, aiming to improve the performance [**11**]. Some researchers believe there are three factors: human agents, organizational gents and technical agents [**12**]. 

In Grader et al.’s research entitled “Evaluating hospital information systems groom nurses’ point of view in medical-educational hospitals of Tabriz”, the highest rate of nurses’ satisfaction regarding the hospital information systems was related to the compatibility with the users’ expectations measure of 2.96, the properness for doing the duty of 2.93, the properness for education of 2.93, standing the error of 2.83, self-description of 2.77, controllability of 2.72, being proper for making it specialized of 2.56 points. The highest rate of the nurses’ satisfaction with Raiavaran company software had a median of 2.95 [**2**]. A research by Saieedbakhsh et al., entitled “The evaluation of the chosen clinic data procedure medical records principal of Isfahan medical university based on 9241-10 standard” showed that the software’s proportion with the employee’s duties was 68 percent. The software’s self-description was 67 percent, user control ability was 70 percent, the compatibility with the users’ needs was 74 percent, the error was 69 percent, the possibility of turning non-governmental was 53 percent, the user’s adequate education was 68 percent, and the user’s acceptance was 68 percent [**1**]. Since that information is a critical element for the effective development and management of health care services, managers of healthcare information systems should regularly monitor these systems and their performances directly, and place their decisions for the development, improving the health systems based on real problems existing in the health care area [**9**]. Analyzing the users’ needs as a valuable tool will increase the users’ satisfaction and lead to an increase in the quality of the medical health care. Identifying the users’ attitude toward systems’ quality and efficiency is critical and is useful in the development and evolution of the system, leading to hospitals’ efficiency and effectiveness, the complications of hospital information systems on one side and the verity of the companies presenting them in Kermanshah Medical University educational-medical centers on the other side. The necessity for a continuous evaluation of these systems to ensure their efficiency in order to satisfy the health systems’ requirements and increase the improvement based on the Ministry of Health’s measures, shows the essentiality of studying and evaluating the hospital information systems from the users’ point of view, them being the ones who use these systems more than anyone in hospitals. The goal to execute the present research was to determine the hospital information systems’ condition based on the Isometric standards in Kermanshah Medical-Educational treatment centers’ medical records users’ point of view during 2014. 

## Method

The present research was applicative and descriptive-sectional with a statistical research society, including 70 individuals and all the users of the medical records of Kermanshah Medical-educational centers during the second half of 2014. All the users of the medical records section of hospital information systems were represented by the research society, and there was no sampling. Among all the employees of the medical records section who used the hospital information system, 64 individuals completed the questionnaire. The tool to gather the data was the 10th part of 9241/ 10 Isometric questionnaire of evaluating hospital information systems. This questionnaire had 75 specific questions including seven measures (proper for doing the duties, self-description, controllability, compatibility with users’ expectations, good for specializing, suitable for learning) which were designed based on 9241 isometric measures. The questionnaire was based on the Likert Scale in a 5-point domain from “entirely improper” to “totally proper”, and “no idea” and a score of 1 to 5 was considered for each question based on its importance. Then a weight was devoted to each subject. Some issues of this questionnaire had a harmful nature and their like scores was reversed, which meant that the point started from 5 to 1 and were applied in the analysis in this manner. The admissibility of the questionnaire’s context was confirmed in previous studies [**1**,**2**]. The permanency of the questionnaire was also established by the Cronbach’s alpha test, which was equal to 0.89. The data were analyzed by using SPSS Software version 22. Descriptive statistics indexes such as distribution redundancy table, two-dimensional tables, number, percentage, numerical indexes such as median and standard deviation and related diagrams were used. K square tests, correlation coefficient, and single way variance analysis were used to evaluate the relation between the primary variants and the personal characteristics (age, gender, marital status, etc.). 

**Findings**

Among the 70 users of the hospital information system of medical records section, 64 individuals completed the 9241/ 10 Isometric hospital information system evaluation standard questionnaire and took part in the research, results showing that among the 64 people who responded to the survey, 57.8% were women, and 42.2% were men, mentioning the users’ academic degree as being the following: 6.2 percent ungraduated, 34.4 high school graduated, 14.1 technicians, 40.6 B.A, and 4.7 percent had levels higher than B.A., 35.9 percent of them had ultimately passed this course, 32.8 passed this course incompletely, and 31.2 did not attend this course at all. 34.4 percent of these individuals passed the CDL course. Finally, 34.4 percent attended it deficiently, and 31.2 percent did not pass this course at all. The median age of users was 35 years and most of the responders (45.3 percent) were in the age group of 3 to 40. 23.4 percent were in the age group of 20 to 29 years, and 31.2 percent were older than 40 years (**Table 1**).

**Table 1:** The distribution of users of medical records section’s attitude in relation with the hospital information systems

Totally proper	proper	No idea	improper	Totally improper	Attitude/Isometric measures
14.69	48.54	14.89	17.81	4.06	Being proper to do the duty
5.34	47.13	22.92	19	5.6	Self-description
8.95	51.56	19.6	17.19	2.7	Controllability
12.09	50.86	16.7	17.08	3.26	Compatibility with users’ requirements
9.48	42.21	20.62	18.75	5.94	Accepting the error
10.31	39.37	20.94	21.56	7.81	Proper for being specialized
7.42	38.87	23.05	27.15	3.51	Being proper for learning
9.92	46.77	19.57	19.32	4.41	Total

Research findings showed that the highest median scores were related to the measure of compatibility with the users’ requirements (3.56) and properness for doing the duty (3.52), and the lowest median scores were related to the measure of being proper for the learning (3.20). Generally, 56.69 percent of the users were satisfied with the hospital information systems and the highest rate of satisfaction respectively belonged to the measure of being proper for the duty (63.23 percent), compatibility with users expectations (62.95) and controllability (60.51), the lowest level of satisfaction belonging to the properness for the learning of 46.29 percent and being proper for being specialized of 49.68. 56.69 percent of the users believed that the hospital information systems are proper and totally proper (**Table 2**-**4**).

**Table 2:** The comparison between the significant level of seven Isometric evaluation elements of information systems and the users’ specifications of medical records section

Individual specifications	gender Satisfaction level				age Satisfaction level			
*isometric measures*	male	female	total	value	Younger than 30	30-40 years old	Older than 40	total	value
Proper for doing the duty	34.26	28.97	63.23	0.000<	20.31	22.51	20.41	63.23	0.7
Self-description	29.28	23.19	52.47	0.000<	17.29	18.89	16.29	52.47	0.000<
Controllability	33.79	26.72	60.51	0.01<	20.67	21.97	17.87	60.51	0.01<
Compatibility with users’ needs	36.96	25.99	62.95	0.4	22.3	21.35	19.3	62.95	0.2
Accepting the error	27.9	23.79	51.69	0.000<	19.89	17.8	14	51.69	0.000<
Properness for being specialized	26.19	23.49	49.68	0.04<	15.93	17.53	16.22	49.68	0.1
Being proper for learning	25.4	20.9	46.29	0.2	15.29	16.54	14.46	46.29	0.06

**Table 3:** Comparing the level of meaningfulness of the satisfaction level of seven measures belonging to information systems isometric evaluation and the individual specialties of the medical records section users’

Isometric measures	Married/ unmarried				education				
	single	married	total	value	Medical records	computer	others	total	value
Properness for doing the duty	15.43	47.8	63.23	0.2	17.72	8.4	36.11	63.23	0.09
Self-descriptive	11.69	40.78	52.47	0.000<	16.19	6.39	29.89	52.47	0.01<
Being controllable	12.8	47.71	60.51	0.000<	17.9	6.9	35.7	60.51	0.2
Computability with users’ expectations	14.13	48.82	62.95	0.01<	20.95	7.4	34.6	62.95	0.5
Accepting the error	12	39.69	51.69	0.000<	15.13	7.24	29.32	51.69	0.05<
Properness for being specialized	10.35	39.33	49.68	0.05<	11.95	6.96	30.77	49.68	0.05<
Properness for learning	10.89	35.4	46.29	0.04<	13.99	5.3	27	46.29	0.4

**Table 4:** Comparison between the significance level with the satisfaction level of seven measures of information systems isometric evaluation and the specializations of the medical records section users’

Individual specialization	education Satisfaction level					Service history Satisfaction level				
Isometric measures	High school graduated or under graduate	Technician and B.A.	M.A. and higher	total	value	Less than 10	10-20	More than 20	total	value
Proper for doing the duty	26.65	33.38	3.21	63.23	0.1	32.41	18.61	12.21	63.23	0.000<
Self-description	20.54	28.33	3.6	52.47	0.03	27.63	13.14	11.7	52.47	0.000<
controllability	23.7	33.81	3	60.51	0.000<	32.32	16.09	12.1	60.51	0.000<
Computability with users requirements	23.81	35.44	3.7	62.95	0.1	35.15	16.15	11.65	62.95	0.03<
Accepting the error	21.44	26.95	3.3	51.69	0.1	30.09	13.3	8.3	51.69	0.1
Proper for being specialized	21.73	24.75	3.2	49.68	0.05<	26.19	11.6	11.89	49.68	0.02<
Proper for learning	16.39	27.2	2.7	46.29	0.2	26.18	10.99	9.12	46.29	0.4

## Discussion

The findings of the present research showed that the highest median scores were related to the measure of computability with users’ requirements (3.56) and properness for doing the duty (3.52), and the lowest median scores were related to the measure of properness for learning (3.20). The total average of all scores was equal to 3.39. The whole 56.69 percent of hospital information systems users were satisfied and the highest level of satisfaction was respectively related to the measure of being proper for doing the duty (63.23), compatibility with users’ requirements or expectations (62.95 percent) and controllability (60.51 percent) and the lowest level of satisfaction was related to the measures of properness for learning (56.29 percent) and properness for being specialized (49.68 percent). 56.69 percent of the hospital information systems users considered this system as proper and totally safe. The difference between the median of seven measures’ score in four companies presenting the hospital information system did not become significant by using the single variance test. 

Users’ satisfaction with the hospital information systems was equal to 53.2 percent in Kimiafar et al. [**13**], equal to 67.5 percent in Saieedbakhsh et al. [**1**] and equal to 64 percent in Gholamhosain et al. [**14**]. The result of the present research (56.69 percent) was similar to other studies’ results. The properness of information systems for doing the duty median measure was equal to 3.04 in Dr. Ahmadi et al. [**15**]. Ghaderi et al. presented a 2.93 percent [**2**], Mousavi et al. highlighted a 2.81 percent [**16**] and, in the research by Hamborg et al., it was equal to 2.7 percent [**8**]. As a result, the current research (3.52) was closer to the results of Dr. Ahmadi et al. in comparison with the other studies. In Saidbakhsh et al., the level of the users’ satisfaction of doing the duty was 68 percent [**1**], in Alipour et al. it was 72.7 percent [**17**] and in Farzi et al. it was 80 percent [**18**]. The result of the present research (63.23 percent) was closer to the results presented by Saiedbakhsh et al. 

About the measure of being proper for doing the duty, the lowest level of users’ satisfaction was related to performing repetitive functions to doing continuous repetitive things by the user (57.9 percent), modulating unrelated duties to the user (57.8 percent), and going through so many stages for doing the duty by the user (53.1 percent). The highest level of the users’ satisfaction was about helping the user complete daily tasks (86 percent) and supporting the executed performances in a software of user’s duties (84.4 percent). In the research by Farzi et al. [**17**], users were not satisfied with going through so many stages to do a task (39 percent). In a research by Saieedbakhsh et al. [**1**], the lowest rate of satisfaction was related to the possibility of quickly finding the required suggestions to do a task by the user (57.6 percent). The highest level of satisfaction was related to the users’ complete comprehension of the fields order on the screen (87 percent). In Ghaderi et al. [**2**], the maximum disagreement among the users with the software was related to modulating the extra work to the user. The present research was similar to Ghaderi et al.’s research. 

The median of users’ scores in self-description measure was equal to 2.86 in Dr. Ahmadi et al. [**15**], 2.77 in Ghaderi et al. [**2**], 2.51 in Mousavi et al. [**16**] and 2.66 in Hamborg et al. [**8**]. The results of the present research (3.33) were closer to the results submitted by Dr. Ahmadi et al. in comparison to other studies. The level of the users’ satisfaction of the self-description measure was equal to 67 percent in Saieed Bakhsh et al. [**1**], 58.9 percent in Alipour et al. [**17**] and 71 percent in Farzi et al. [**18**]. The results of the present research (52.47 percent) were closer to Alipour et al.’s. Regarding the self-description measure, the level of the users’ satisfaction of giving general descriptions and real examples with visual points to the user in case of the requirement was equal to (40.6 percent). The lack of giving required explanations to the user regarding the way he should use the system was equal to 39 percent. The maximum level of satisfaction of understanding the given messages immediately by the user and showing the condition of entering the input on the software (for example by contrasting colors, crosser turning on and off, highlighting and others) was equal to 68.7 percent. 40 percent of the users were dissatisfied in Farzi et al.’s [**18**] research about understanding the visual issues that are out of reach. 42 percent were dissatisfied in the case of not showing the general descriptions and real examples with visual points to the user in the event of need. In a research by Saieedbakhsh et al. [**1**], the lowest level of satisfaction with showing the general explanations and real examples with visual points to the user in case of need (51.2 percent) and the maximum level of satisfaction with immediate comprehension of messages shown on the screen by the users were equal to 81.2 percent. In a research by Ghaderi et al. [**2**], the maximum level of users’ agreement was with the quick realization of the presented messages on the screen, and the highest level of disagreement was with the lack of showing real examples with visual points. The results of the current research were similar to the ones mentioned. 

The users’ median scores about the measure of being controllable was equal to 3.09 in Dr. Ahamdi et al. [**15**], 2.27 in Ghaderi et al. [**2**], 2.63 in Mousavi et al. and 3.03 in Hamborg et al. [**8**]. The results of the present research (3.47) were closer to the results presented by Dr. Ahmadi et al. and Hamborg et al. in comparison with other studies. The level of users’ satisfaction with the masseur of being controllably was equal to 70 percent in Saieedbakhsh et al. [**1**], 76.9 percent in Alipour et al. [**17**] and 57 percent in Farzi et al. The results of the present research (60.51 percent) were closer to the ones of Farzi et al. 

About the measure of being controllable, the lowest level of users’ satisfaction was related to the lack of ability in the help center existing software and the lack of support in the software’s guiding center with regard to the system’s applicative functions (29.7 percent). The highest level of satisfaction was respectively related to the possibility of natural movement of the user among the previous and the next pages of the screen (76.6 percent), the obligation of the user to do a series of permanent steps to do his/ her tasks with the help of the software (70.3 percent) and the possibility of speeding up the selection of menu subjects by directly entering a proper letter for learning a code (68.8 percent). In Farzi et al. [**18**], 38 percent of the users were dissatisfied about the easy understanding of each dialogue, each time. In a research by Saieedbakhsh et al. [**1**], the lowest level of satisfaction was related to the possibility of speeding up the selection of a subject from the menu with the direct entering of the code (62.4 percent) and the maximum level of satisfaction was related to the possibility of the user’s natural movement between the previous and the next pages (85.8 percent). The highest percent of disagreement was related to the lack of existence of enough guides in the system, as presented in the study of Ghaderi et al. [**2**]. The results of the present research were compatible with the ones in the previous studies. 

The median users’ scores to measure the compatibility with the users’ expectations were equal to 3.14 in Dr. Ahmadi et al. [**15**], 2.92 in Ghaderi et al. [**2**], 2.65 in Mousavi et al. [**16**] and 3.13 in Hamborg et al. [**8**]. The results of the present research (3.56) were closer to the ones of Dr. Ahmadi et al. in comparison with other studies. The level of the users’ satisfaction was equal to 74 percent in Saieedbakhsh et al., 73.7 percent in Alipour et al. [**17**], 61 percent in Farzi et al. [**18**]. The result of the present research (62.95) was closer to the one of Farzi et al. 

In relation to the measure of compatibility with the users’ expectations, the lowest level of users’ satisfaction was about the difficulty of doing the tasks by the user, due to the lack of stability in designing the software (46.9 percent). The highest level of satisfaction was with the inherent possibility of predicting the required time for doing the tasks in the software (73.5 percent) and the possibility of predicting the displayed screens in the next level by the user (73.4 percent). In a research by Farzi et al. [**18**], 27.8 percent of the users were dissatisfied in relation to the difficulty of doing the job, due to the lack of stability in designing the software. In a research by Saieedbakhsh et al. [**1**], the lowest level of satisfaction was in relation to the balance of concepts used in all the parts of the software and the difficulty of doing the tasks, due to the lack of confidence in designing the software (62.6 percent) and the maximum level of satisfaction was in relation to the possibility of predicting the displayed pages in the next level by the user (89.4 percent). In a research by Ghaderi et al. [**2**], the maximum item that most users agreed upon was the ability to predict the next page and, the most fundamental problem in relation to this, from the users’ point of view, was the lack of stability of the concepts in all the parts of the software. The results of the present research were similar to the ones in the other studies.

The median users’ score in relation to the measure of accepting the error was equal to 2.95 in Dr. Ahmadi et al. [**15**], 2.83 in Ghader et al. [**2**], 2.61 in Mousavi et al. [**16**] and 2.72 in Hamborg et al. [**8**]. The results of the present research (3.34) were closer to the results presented by Dr. Ahmadi et al. in comparison with other studies. The level of users’ satisfaction regarding the measure of accepting the error was equal to 69 percent in Saieedbakhsh et al. [**1**], 53.7 percent in Alipour et al. [**17**] and 75 percent in Farzi et al. [**18**]. As a result, the present research (51.69 percent) was closer to the one of Alipour et al.

The median users’ score in relation to the measure of accepting the error was equal to 2.95 in Dr. Ahmadi et al. [**15**], 2.83 in Ghader et al. [**2**], 2.61 in Mousavi et al. [**16**] and 2.72 in Hamborg et al [**8**]. The results of the present research (3.34) were closer to results presented by Dr. Ahmadi et al. in comparison with other researches. The level of user’s satisfaction with the measure of accepting the error was equal to 69 percent in Saieedbakhsh et al. [**1**], 53.7 percent in Alipour et al. [**17**] and 75 percent in Farzi et al. [**18**]. As a result, the present research (51.69 percent) was closer to the one of Alipour et al. 

Regarding the measure of accepting the error, users showed the minimum level of satisfaction towards the probability of severe consequences resulted from minor mistakes while working with the software (50 percent), wasting so much time on the software before noticing the wrong data (45.3 percent), lacking a presentation of a system error (such as twists) while working with the software (36 percent) and the maximum level of satisfaction was related to the request for a user’s confirmation of disposal functions (such as eliminating the data) (70.3 percent). In a research by Farzi et al. [**18**], 39 percent of the users were satisfied in relation to severe consequences due to minor mistakes made by the user, 45 percent of the users were satisfied regarding the delay in announcing the wrong input and 36 percent of the users were dissatisfied with the lack of presenting a system error while working with the software. In the research of Saieedbakhsh et al. [**1**], the lowest level of satisfaction was with the lack of presenting the system error (such as a twist) while working with the software (49 percent) and the maximum level of satisfaction was related to the request for a user’s confirmation of disposal functions (such as eliminating the data) (87 percent). 

In a research by Ghaderi et al. [**2**], the item with highest level of disagreement was the system error (the locking problem) that happened while working with the system. The results of the present research were in agreement with other studies. The median users’ scores for the measure of properness for being specialized was equal to 2.57 in Dr. Ahamadi et al. [**15**], 2.56 in Ghaderi et al. [**2**], 2.39 in Mousavi et al. [**16**] and 2 in Hamborg et al. [**8**]. The results of the present research (3.23) were not close to the results of the other studies. The level of satisfaction of the users with the measure of properness for being specialized was equal to 53 percent in Saieedbakhsh et al. [**1**], 66.3 percent in Alipour et al. and 72 percent in Farzi et al. The results of the present research (49.68 percent) were the closest to the results presented by Saieedbakhsh et al. 

Regarding the measure of properness for being specialized, the minimum level of satisfaction of users was related to the lack of possibility to change the title of orders and subjects and tasks based on the users’ word collection (37.5 percent) and the lack of the possibility of changing the forms, display screens and menus based on desire (34.4 percent), the maximum level of satisfaction being related to the possibility of setting the amount of displayed information on the screen, based on the user’s needs (59.4 percent), an option to quickly adjust the software proper to the user’s level of skill and knowledge (57.8 percent). In a study by Farzi et al. [**18**], 36 percent of the users were dissatisfied in relation to the permission of changing the forms, screenplays, and menus based on the user’s desire and 31 percent were dissatisfied with setting the response times of the software with the required speed. In a research by Saieed Bakhsh et al. [**1**], the minimum level of satisfaction was related to the possibility of changing the forms, screen and menus as desired by the user (42.4 percent) and the maximum level of satisfaction was related to the possibility of quickly adjusting the software based on the user’s skill and knowledge level (68.2 percent). In a research by Ghaderi et al. [**2**], the most significant problem from the users’ point of view was the lack of the software’s response time adjustment with the users’ speed. The results presented in the research were similar to the ones in other studies. The users’ median score of the measure of being proper for learning was equal to 2.97 in Dr. Ahmadi et al. [**15**], 2.93 in Ghaderi et al. [**2**], 2.70 in Hamborg et al. and 2.70 in Mousavi et al. [**16**]. The results of the present research (3.20) were closer to Dr. Ahmadi et al. more than to others. 

The level of the users’ satisfaction in the measure of being proper for learning was equal to 68 percent in Saieedbakhsh et al. [**1**], 58.9 percent in Alipour et al. [**17**] and 79 percent in Farzi et al. The results of the present research (46.29 percent) were closer to the results submitted by Alipour et al. In relation to the measure of being proper for users to learn, the lowest level of users satisfaction was about the long time period required for the user to work with the system (59.4 percent) and the necessity of remembering so many details by the user for the proper use of the software (50 percent) and the maximum level of satisfaction was related to the possibility of natural re-learning of system usage in case they did not use the system for a long time (73.4 percent). In the research by Farzi et al. [**18**] about needing to remember so many details for the correct use of the software, dissatisfaction was 25%. Moreover, 38% were dissatisfied with the right way to use the software from the beginning. In a research by Saieedbakhsh et al. [**1**], the lowest level of satisfaction was with the long time required for learning the system (48.2 percent) and the maximum level of satisfaction was related to the possibility of natural re-learning of how to use the system in case it was not used for a long time (85.8 percent). In a research by Ghaderi et al. [**2**], the maximum percentage of the users’ agreement was related to easy re-learning of how to use the system in case it was not used in a long time. The results of the present research were similar to the ones in other studies. 

Based on the results of the current research, the users who passed their HIS course completely had the highest level of satisfaction with regard to each seven measures and the users who passed their ICDL course completely had the highest degree of satisfaction of the 7 tests but for the measure of properness for being specialized. In all the seven measures, users who announced their computer skills to be right or high, had the highest rate of satisfaction. Users with a higher computer education and training related to the appliance of HIS had the highest level of satisfaction with the system. The critical point of educating users before using the system, time of starting it and of executing the system at all levels led to the deeper and quantitative understanding of the system and of all flaws in hospital information system, leading at the end to its optimized use. 

The relation between the users’ individual characteristics and their level of satisfaction with hospital information systems seven-measure evaluation was studied by using a test. There was a significant relationship between the users’ gender and the proper measures for doing the task, controllability, accepting the error and the properness for being specialized. Male users were more satisfied with these measures. There was a significant relation between the age and the level of satisfaction with measures of self-description, controllability, and accepting the error. Older age ranges showed lower levels of appreciation. There was a significant relation between the users’ marital status and the level of their satisfaction with the seven measures, but not for the action of being proper for doing the task. The married users were more satisfied than single ones. The users’ academic branch and their satisfaction with measures to evaluate the hospital information systems in measures such as self-description, accepting the error and properness for being specialized had a significant relation. The level of satisfaction in users with academic degrees of other branches but for medicine and computer was higher. The relationship between the users’ educational degree and their level of satisfaction of evaluating measures was significant regarding the means of self-description, controllability, and properness for being specialized. The users with academic degrees of college and B.A. had the highest rate of satisfaction in comparison with the others. There was a significant relation between the level of the users’ service history and the level of satisfaction with all measures but for accepting the error and being proper for learning. As the service history increased, the satisfaction with the measures decreased. In a research by Gholamhoseini et al., 100 percent of the users in the medical records section were satisfied with using the hospital information system [**14**], which was not similar to the results of the present research. 

## Conclusion 

The evaluations of the hospital information system up until now, mostly concentrate on financial aspects, the patients’ interests and the important points. What they have been neglecting was that they did not notice the users’ attitude and ability to use the system by nurses, doctors and other employees and people who spend a significant part of their day on this software [**8**]. If a good information system was described as a weak system for a user, that system will be small [**19**]. The human parameters’ role in executing and employing computer systems could advance these procedures’ capability [**20**]. 

The results of this research showed that the users of medical records of educational–medical centers showed the highest rate of satisfaction towards the measure of being proper for doing the task (63.23) and the compatibility with the users’ expectations (62.95) and the lowest level of satisfaction was towards the measures of being proper for learning (46.29 percent). In designing the hospital information systems, a low level of attention has been paid to the measures of being useful for learning and specializing in the operation by the user. 

Among the most important measures of the ability to privatize and being easy to know for the user we could mention items such as the capacity to change forms, screen and menus based on the users desire and by his/ her software’s compatibility with the users knowledge and skill level, thus adjusting the level of displayed information on the screen. The possibility of changing the title of orders and objects and actions, proper with the users’ personal word collection, helping by the presented descriptions to the user to (or “intending to”) create greater skills in using the software, lack of problems in learning the rules related to the use of software by the user. The possibility of a correct use by the user without asking colleges for help, encouraging users to buy the software to test the preferred functions related to a new system by testing and the essentiality of remembering so many details by the user to use the software correctly can be mentioned among them. In the present research, users were least satisfied with the lack of possibility to change orders, subjects and actions based on the user word collection and lack of opportunity to change forms, the screen and menus as desired by the user, the long time required for learning the use of the system and the necessity to remember many details by the user to use the system and menus correctly. In most researches, users were least satisfied by these aspects. 

In other measures, the highest rate of dissatisfaction was observed for repetitive actions to do continuous works by the user, positioning unrelated tasks to the user, going through so many stages to do a task, lack of playing the general information on the screen and real examples with visual notions if necessary, lack of presenting the necessary explanations to the user for using the system, the poor performance of the existing software and lack of support from the software help center as the practical function of the software, difficulty in doing the tasks by the user due to the lack of stability in designing the software, the possibility of severing consequences due to minor mistakes while working with the software, wasting so much time on the software before noticing the wrong data and system errors (getting twisted) while working with the software were observed, which were similar to the previous results. 

The results of the present research showed that users of hospital information systems were satisfied or rather satisfied in most aspects but for being privatized from being proper for learning, considering the long distance between the existence of information systems and the lack of desired performance. It is essential that they pay more attention to their movement and development and find a deeper understanding of the users’ opinions and needs to increase their success chances to achieve their goal, improve the level of patient care and the individuals’ health in society by using information technology [**21**]. Thus, paying attention to the various aspects presented in the isometric questionnaire is critical to design these systems. 
